# Supplementary material for: From sequence to enzyme mechanism using multi-label machine learning
Source: BMC Bioinformatics. 2014 May 19;15:150. doi: 10.1186/1471-2105-15-150 (PMC4229970; doi:10.1186/1471-2105-15-150)
Supplement: Additional file 2 — Java code of ml2db. Additional file ml2db_code.tar.gz contains the Java source code to run the multi-label machine learning experiments and save the results to database. The code’s Javadoc is included. [file 1471-2105-15-150-S2.zip › additional file 2/ml2db/ml2db/doc/main/ml2db/mulan/learn/crosseval/CrossEvaluator.html]

CrossEvaluator


JavaScript is disabled on your browser.


- Overview
- Package
- Class
- Use
- Tree
- Deprecated
- Index
- Help

- Prev Class
- Next Class

- Frames
- No Frames

- All Classes

- Summary:
- Nested |
- Field |
- Constr |
- Method

- Detail:
- Field |
- Constr |
- Method


main.ml2db.mulan.learn.crosseval

## Class CrossEvaluator

- java.lang.Object
- - mulan.evaluation.Evaluator
  - - main.ml2db.mulan.learn.evaluation.EvaluatorNoAUC
    - - main.ml2db.mulan.learn.crosseval.CrossEvaluator

- Direct Known Subclasses:
  :   TrainTestEvaluator

  ---

    

  ```
  public class CrossEvaluator
  extends EvaluatorNoAUC
  ```

  Class created to execute cross-evaluations and extract individual evaluation
  results (not possible from the Mulan class Evaluator, because only averaged
  results are returned). Calls `ResultsOutput` to save the results to
  database.

  Version:
  :   8 Jun 2010

  Author:
  :   Luna De Ferrari ldf(at)st-andrews.ac.uk

- - ### Constructor Summary

    Constructors

    Constructor and Description

    `CrossEvaluator(CrossExperimenter experimenter,
    boolean strict)`
  - ### Method Summary

    Methods

    | Modifier and Type | Method and Description |
    | --- | --- |
    | `java.util.Vector<java.lang.Object[]>` | `evaluate(int numFolds, IdMultiLabelInstances trainDataSet, mulan.classifier.MultiLabelLearner learner, boolean savePredictions, boolean saveTrueNegatives, boolean stratify)` Evaluates (runs a cross validation) |
    | `java.util.Vector<java.lang.Object[]>` | `internallyCrossValidate(int numFolds, int seed, IdMultiLabelInstances trainDataSet, mulan.classifier.MultiLabelLearner learner, boolean savePredictions, boolean saveTrueNegatives, boolean stratify)` Evaluates a `MultiLabelLearner` via cross-validation on given data set with defined number of folds. |

    - ### Methods inherited from class main.ml2db.mulan.learn.evaluation.EvaluatorNoAUC

      `evaluate, evaluate, getEndTime, getExperimenter, getStartTime, getStrictness, isStrict, setEndTime`
    - ### Methods inherited from class mulan.evaluation.Evaluator

      `checkData, checkLearner, crossValidate, crossValidate, evaluate, evaluate, setSeed`
    - ### Methods inherited from class java.lang.Object

      `equals, getClass, hashCode, notify, notifyAll, toString, wait, wait, wait`

- - ### Constructor Detail


    - #### CrossEvaluator

      ```
      public CrossEvaluator(CrossExperimenter experimenter,
                    boolean strict)
      ```
  - ### Method Detail


    - #### evaluate

      ```
      public java.util.Vector<java.lang.Object[]> evaluate(int numFolds,
                                                  IdMultiLabelInstances trainDataSet,
                                                  mulan.classifier.MultiLabelLearner learner,
                                                  boolean savePredictions,
                                                  boolean saveTrueNegatives,
                                                  boolean stratify)
      ```

      Evaluates (runs a cross validation)

      Parameters:
      :   `mlDataSet` -
      :   `numFolds` -


    - #### internallyCrossValidate

      ```
      public java.util.Vector<java.lang.Object[]> internallyCrossValidate(int numFolds,
                                                                 int seed,
                                                                 IdMultiLabelInstances trainDataSet,
                                                                 mulan.classifier.MultiLabelLearner learner,
                                                                 boolean savePredictions,
                                                                 boolean saveTrueNegatives,
                                                                 boolean stratify)
                                                                   throws java.lang.Exception
      ```

      Evaluates a `MultiLabelLearner` via cross-validation on given data
      set with defined number of folds. The specified number of folds has to be
      at least two. If negative value is specified, the used number of folds is
      equal to number of instances in the data set. Also saves the data to file
      and database.

      Parameters:
      :   `numFolds` - the number of cross evaluation folds (n)
      :   `seed` - a random seed for randomising instances into n folds
      :   `trainDataSet` - the training set
      :   `learner` - the algorithm
      :   `savePredictions` - whether predictions should be saved to database
      :   `stratify` - whether the folds should be stratified

      Returns:
      :   an array containing: an integer to determine the nth fold (as
          String), startOfRun time (String), endOfRun time (String), an
          Evaluation object containing the evaluation metrics
          (mulan.core.Evaluation)

      Throws:
      :   `java.lang.Exception`


- Overview
- Package
- Class
- Use
- Tree
- Deprecated
- Index
- Help

- Prev Class
- Next Class

- Frames
- No Frames

- All Classes

- Summary:
- Nested |
- Field |
- Constr |
- Method

- Detail:
- Field |
- Constr |
- Method
